# The German Young Olympic Athletes' Lifestyle and Health Management Study (GOAL Study): design of a mixed-method study

**DOI:** 10.1186/1471-2458-11-410

**Published:** 2011-05-31

**Authors:** Ansgar Thiel, Katharina Diehl, Katrin E Giel, Alexia Schnell, Astrid M Schubring, Jochen Mayer, Stephan Zipfel, Sven Schneider

**Affiliations:** 1 Institute of Sport Science, University of Tübingen, Tübingen, Germany; 2 Mannheim Institute of Public Health, Social and Preventive Medicine Medical Faculty Mannheim, Heidelberg University, Mannheim, Germany; 3 Department of Psychosomatic Medicine and Psychotherapy, Medical University Hospital, University of Tübingen, Tübingen, Germany

**Keywords:** health management, elite sports, adolescent athletes, health promotion, health behavior, lay health representations, attitude to health, subjective health state, health cultures, risk behavior, risk-taking, eating disorders, self-medication, mixed-method, social networks

## Abstract

**Background:**

In order to perform at top levels, elite athletes have to both protect and risk their health at the same time. Adolescent elite athletes have the additional challenge of coping with substantial physical, psychological and social transformations. The contradictory phenomenon of protecting and risking the adolescent athletes' health in sports challenges the development of health promotion and protection strategies. The GOAL Study (German Young Olympic Athletes' Lifestyle and Health Management Study) analyzes the individual and organizational management of health in adolescent elite sports.

**Methods/design:**

We combine quantitative and qualitative approaches in a mixed-method study. This allows us to gather a broad range of representative information on squad athletes from all Olympic disciplines as well as in-depth information on four selected Olympic disciplines (artistic gymnastics, biathlon, handball and wrestling). Within the quantitative section we attempt to identify the young athletes' health and nutrition behavior, their subjective health state and their lay health representations, health-related social networks, and structures of medical attendance. 1138 national team level athletes born between 1992 and 1995 from 51 Olympic disciplines responded to the questionnaire (response rate: 61,75%). The qualitative section investigates the meaning and relevance of health and nutrition within the athletes' sports specific surroundings, the impact of biographic backgrounds on individual health behavior, and sports specific cultures of health, nutrition and risk. We interviewed 24 athletes and 28 coaching and medical experts, and carried out 14 multi-day participant observations at training sessions and competitions.

**Conclusions:**

The studies' results will serve as the basis for developing tailored health promotion strategies to be in cooperation with German elite sports associations.

## Background

Competing at top levels causes athletes to risk their health by pushing their physical and mental limits, both in training and competition. Difficulties may arise in managing potential health risks such as overtraining, malnutrition, drug abuse and playing hurt. In competitive sports, good health is the necessary foundation for developing peak athletic performance. This phenomenon of protecting and risking the athletes' health challenges the development of health promotion and protection strategies, especially for young athletes [1,2].

Adolescents are normally hardly concerned with their health. At the same time they have to cope with substantial physical, psychological and social developments. Considering this sensitive phase of development it is therefore imperative to provide young elite athletes effective structures for promoting a healthy and successful sports career.

Based on constructivist approaches [3-6], we consider individuals' representations of their social world, their behavior and biographical developments as a complex interplay between themselves and their different social systems. Against this background, we assume that subjective health concepts and theories as summarized in the expression 'lay health representations', largely influence individuals' health-related behavior and subjective health state [7-9].

The lay health representations of athletes are generally influenced by their social networks (e.g., general conditions of their sport or their sports associations' medical treatment offers) and shaped by significant life events which may lead them to focus on specific health-related topics like prevention or nutrition. Thus, the athletes' lay health representations and the resulting health-related behavior are subject to ongoing changes over the course of their career [2].

Most studies concerning the young athletes' health deal with sports specific injuries and illnesses [10-12] and health-related behaviors such as disordered eating [13], (recreational) drug use [14] and dietary supplement use [15,16]. Additionally some of these studies examine the athletes' attitudes towards specific behaviors like mouthguard use [17], doping and drug abuse [18], or competing in pain [19] Yet, young elite athletes' individual health management, seen as their overall and sports-specific health-related behavior, is still not thoroughly examined. While there are a lot of representative studies dealing with subjective health state, health complaints and risky behavior in adolescents [20,21], a comparison with values of young elite athletes is not possible until now.

Considering the increasing number of sociological studies address the effects of elite sports specific risk cultures on managing pain and injury [22-26], almost nothing is known about the characteristics and mediation of certain risk cultures in youth elite sports and its impact on health promotion or injury prevention strategies. Explorative approaches on elite athletes' accounts on health [27] or lay health representations [2] show the significance of career socialization processes in developing a sports specific understanding of threats, health resources and the meaning of future well-being. Likewise, little is understood about the adolescent athletes' lay health representations, its significance for healthy behavior and its development under the influence of formal and informal support systems and social environments. Therefore, still to be thoroughly examined is the role of peers, parents, coaches, healthcare providers, sports associations, and other significant support systems within adolescent elite sports' health protection and promotion processes. Until now, there is alack of health promotion and protection strategies for adolescent elite sports, which are empirically based and developed under participation of the relevant sports associations.

### Research questions and methodological approach

**Figure 1:**
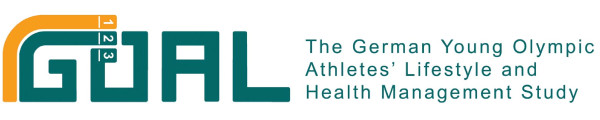
**Logo GOAL Study**.

The **G**erman Young **O**lympic **A**thletes' **L**ifestyle and Health Management Study (GOAL Study; Figure 1) has two central aims: First, to fill the above-mentioned research gaps and second, to develop sports-specific health protection and promotion strategies. In order to create a holistic picture of managing health in German adolescent elite sports, we carried out a nationwide mixed-method study. Combining quantitative and qualitative approaches allowed us to gather a broad range of representative information on squad athletes of all Olympic disciplines as well as in-depth information on four Olympic disciplines: artistic gymnastics, biathlon, handball and wrestling.

Within the quantitative section of the GOAL Study, we attempt to identify the impact of lay health representations (including subjective concepts about nutrition as well) of adolescent athletes, social networks and socio-demographic variables on athletes' health-related behavior and subjective health state on a representative basis.

We aim to answer the following questions within the quantitative study part:

• What kind of lay representations of health do young German Olympic athletes have, and are there any differences related to discipline, age, sex or social background?

• To what extent is the athletes' health-related behavior and subjective health state influenced by lay health representations?

• What health- and especially nutrition-related conflicts are found between young athletes and significant others of their social networks?

• To what extent do health-related behavior and subjective health state differ between adolescent elite athletes and non-athletes?

The qualitative section of the GOAL Study investigates how young athletes, depending on their biographic backgrounds, construct the meaning and relevance of health and nutrition within their sports-specific surroundings. Furthermore, the qualitative analysis allows us to identify sports-specific health cultures and helps us to reconstruct the influence of organizational structures on managing health-related aspects.    

Thus, the following questions are the focus of the qualitative study section:

• How do adolescent athletes deal with health and nutrition, and how is health embodied in their everyday life?

• How do biography and social context influence health and nutrition practices within adolescent elite sports?

• Do sports disciplines differ concerning their health and risk cultures, and if yes, how do these cultures influence the young athletes' health and nutrition behavior?

The studies' results will serve as the basis for tailored health promotion strategies to be developed in cooperation with representatives of German elite sports' associations.

## Design and Methods

### Design, participants and selection criteria

#### Quantitative study

Within the nationwide quantitative study section, we aimed to conduct a complete survey of all young German adolescent elite athletes. Data collection took place between February 2010 and January 2011.

The inclusion criteria were the following: First, participants had to play one of the Winter Olympics 2010 or the Summer Olympics 2012 sports. Second, participants had to be born between the years 1992 and 1995. Third, participants had to compete at least at the lowest national squad (in Germany D/C squad) or a corresponding team level.

In two of the 54 Olympic sports, namely, sailing and bobsledding, no athlete met these criteria. Greco-Roman Wrestling did not agree to partake in the study. Therefore, based on the afore-mentioned inclusion criteria, a total of 1843 athletes from 51 sports were eligible for study participation.

#### Qualitative study

When considering the highly diversified demands and training structures of Olympic sports, we chose a multi-case study approach and focused on four of the Olympic disciplines: artistic gymnastics, biathlon, handball and wrestling.

Based on earlier research [2,28,29], we identified the following criteria to guide our sampling strategy within Olympic sport: (1) discipline's profile (team vs. individual sport; indoor vs. outdoor sport), (2) requirement profile (criteria for scouting, promotion, and acceptance on the national team, participation in national competitions, training guidelines, weight classes), (3) general injury and illness profile, and (4) gender profile. We also looked at feasibility and governing associations' approval to participate in the qualitative study as deciding factors in determining which four disciplines to examine. Seeking information-rich cases, we used maximum variation sampling of these criteria.

Within the four disciplines, we opted for an embedded approach, which allowed us to analyze the athletes' thinking and behavior as well as their social environment structures. We included two different actor types: adolescent elite athletes (I) and different experts (II) from athletes' supportive environment. The participating athletes had to fit the same inclusion criteria as the quantitative sample. We selected experts in regard to their functional role within their sports associations. We also conducted participant observation at training sessions and competitions to provide meaningful insights.

We recruited a total number of 24 German elite athletes (12 females and 12 males) for the qualitative study, aged between 15 and 18 at the time of the participation entry. The athletes were equally distributed across disciplines: biathlon (6), gymnastics (6), handball (6), and wrestling (6). In addition to the 24 athletes, we recruited 28 experts from the participants' social environment, assuring that each defined role within our sampling scheme was covered. In biathlon, the total number of experts is half that of the other disciplines since biathletes of the sampled age group are not gender differentiated within the national training structure.

Most of the experts (16) are men, as no female experts held functional roles on male teams. However, this gender proportion corresponds with the general underrepresentation of women within leading positions in the German sports associations [30].

### Research governance and ethics

The GOAL Study is an interdisciplinary cooperative project funded by the Federal Institute of Sport Science (BISp) in Bonn, Germany. The study's project partners are the University of Tübingen's Institute of Sport Science and Department of Psychosomatic Medicine and Psychotherapy, and the Mannheim Institute of Public Health, Social and Preventive Medicine at Heidelberg University. The Medical Faculty of Tübingen ethics committee's approved the research project (222/2009BO1). As required by the ethics committee, we received written informed consent from each participant, allowing his or her data's inclusion in the study.

### Central topics and data collection techniques - quantitative study

#### Development of the questionnaire

The questionnaire topics based on the aforementioned theoretical assumptions and research questions. Wherever possible, we included already existing, validated subscales or items into the questionnaire. Additionally, our research group chose field-tested scales and items developed and successfully used in previous surveys. If no previous work was available, scales and items were custom-developed for the present questionnaire.

#### Central topics

The participants filled in a 24-page bound questionnaire consisting of 85 questions. These questions covered the athletes' health state, health-related behavior, lay health representations including subjective concepts toward nutrition as well as their health-related social networks, socio-demographics, and discipline-specific information.

#### Health state

In order to obtain a general view of the athletes' subjective health state, we also included questions about their physical, mental and social health state. The physical health state addressed sports-related physical pain and any current medical treatment. The questions as to if the athletes were currently injured or ill, and whether they suffered injuries and diseases during the last season were intended to check the individual's time off due to health concerns. Since the phenomenon "doctor hopping" seems to be a common practice within elite sports, we asked participants about the number of physicians consulted for the same injury.

Previous research revealed several psychosocial problems among elite athletes. To see if this problem is present among different disciplines of German elite athletes, we included questions pertaining to the athletes' psychosocial health state into the questionnaire. Since the level of social role completion influences an athlete's psychosocial health state we first asked to which extent the young athletes focused on their role as elite athletes. We followed up this question by asking if they have alternative, non-sports-related goals that could be a source of satisfaction in the case of long-term competitive failure or injuries.

We also screened participants for burnout (Athlete Burnout Questionnaire, German version [31]), depression (Patient Health Questionnarie-2 (PHQ-2) [32]) and overall perceived stress concerning school, sports, family, and friends. Additionally, our research team screened for eating disorders using the SCOFF instrument [33].

In order to check for symptoms of Female Athlete Triad, the study survey included questions concerning BMI, menorrhea and contraception. In this context, we asked the athletes about their willingness to follow a healthy diet and about their options to choose their own food. Previous research revealed that the risk for eating disorders is associated with a high degree of perfectionism, so we included a perfectionism scale into the questionnaire (Eating Disorder Inventory-2 (EDI-2) [34]), and we screened for body dissatisfaction (Frankfurter Körperkonzeptskalen, (FKKS) [35]). Body image is an integral part of the self-concept and is an especially relevant concept during adolescence because this is often an emotionally vulnerable phase due to the body's constant changes. Body dissatisfaction is also discussed as a risk factor for eating pathology; however, physical exercise might help prevent or fight against this dissatisfaction.

Lastly, we measured sleeping habits both in everyday life and right before a competition as an alternative indicator for overall psychological stress.

#### Health-related behavior

To evaluate the athletes' health-related behavior, we examined both common lifestyle attitudes and sports-specific health-related behavior. Using standardized questions we asked about alcohol consumption [36], binge drinking [37] and smoking habits [36], including the age of onset [38]. We examined nutrition habits using in parts the established field-tested food-frequency scale from the German Health Interview and Examination Survey for Children and Adolescents (KiGGS, [36]). Also included in the survey were the locations where the athletes ate most of their meals, whether they followed an established diet plan, and if dieting behavior existed over the last year. Furthermore, we examined sports-specific health-related behavior such as dietary supplement use, medication, regeneration as well as sports-specific injury and illness prevention. Besides we asked for weight control practices (Strukturiertes Inventar für Anorektische und Bulimische Störungen (SIAB-S) [39]).

#### Cognitive conditions: lay health representations of adolescent elite athletes - subjective health concepts and theories

In order to understand the reasons for health-related behavior, we analyzed the athletes' health representations, meaning subjective health concepts including ideas about nutrition, subjective health theories, and the athletes' willingness to take sport-related risks. Against the background of being highly relevant to elite sports, we also focused on the athletes' general concepts about dietary supplements and the perceived barriers of eating a healthy diet.

#### Social network: significant others, social support and social pressure

Subjective concepts are influenced, to a great deal, by the surrounding circumstances. Thus an adolescent athlete is surrounded by many people belonging to different social systems, thereby representing varied interests. Therefore, the response to the research question of what extent specific people influence the athletes, should be found through examining their social networks. Survey items covered the athletes' social support network, their medical network, and the network used to acquire dietary supplements. Furthermore, we asked the participants about which information sources they consulted regarding health, nutrition, dietary supplements and performance enhancement possibilities. Since the target group is at a vulnerable age of life, we asked the athletes who they contacted in times of distress. In order to assess the athletes' freedom to make decisions, we included questions about who makes competition-related decisions and questions asking the athletes to describe their coach's leadership style and whether anyone controlled their diet. Additionally, we examined if someone expected the athletes to adhere to contractual commitments such as taking specific dietary supplements or abstaining from smoking, following a diet plan, maintaining or gaining a certain amount of weight or attending specific sports medical examinations. Lastly, we asked the athletes for health-related service offers they use while practicing at an Olympic support center ("Olympiastützpunkt").

#### Socio-demographics and discipline-specific information

In addition to general socio-demographics such as age, sex, school grade, residence, and language spoken at home, we requested discipline-specific socio-demographic information including in which discipline they compete, at what age the athlete first was accepted onto the squad and the main training location. We asked about training and competition load, previous engagement in another competitive sport and if family members are or were involved in high performance sports. Two questions invited the athletes to tell us any perceived opportunities to improve their health management.

#### Three-step pretest

We conducted a three-step pretest to improve the questionnaire's quality and to alert our research team to any potential problems that could arise while filling it in. First, we asked twelve female and ten male adolescent athletes to fill in our questionnaire. All were members of competitive swimming or volleyball squads and practiced between three and six times a week, participated regularly in competitions, and had at some point been part of a state-level squad. Second, we asked eight additional athletes to further adjust the questionnaire. A researcher attended each pretest session. To detect any possible misunderstandings, the participants were instructed to ask a researcher to explain anything and everything they did not understand. The researcher noted each question and comment as well as how long it took each athlete to complete the questionnaire. The third step differed from the preceding steps in that we invited the project advisory board, not youth athletes, to comment on the questionnaire. The advisory board consists of German experts from different sports-related fields. After each step, the given comments and identified problems were included in a new version of the questionnaire. We ended the pretest after three steps because no relevant comments or misunderstandings remained.

### Administration

The quantitative study questionnaires were accompanied by personal letters for the athletes and their parents, a two-sided document explaining the study, a consent form, and a prepaid self-addressed envelope. We distributed the questionnaires via different distribution channels: 35 associations requested direct mailing and provided the athletes' addresses, which allowed follow ups by mail. 17 associations opted for central distribution, either as forwarded postal reshipments or to be distributed at squad's central training sessions by trainers and coaches. The questionnaire distribution took place between February 2010 and January 2011. The large time frame was due to the varying training schedules or squad establishments within the disciplines and some athletes' absence during the season. This tailored distribution design and large time frame allowed for the inclusion of a total sample comprised of German elite athletes from our target Olympic sports born between 1992 and 1995.

### Response rate

The overall response rate was 61,75% (n = 1138), calculated according to the standard definitions set by the American Association for Public Opinion Research [40]. The discipline-specific response rates differed according to the method of distribution (Table 1). We carried out non-responder analysis on the questionnaires delivered via direct distribution (Table 1) and found that an equal proportion of male (37.8%) and female (37.2%) athletes did not respond. However, there was a difference in response by birth year: While the non-response rate for the birth years 1993 to 1995 was between 35.5% and 37.5%, the non-response rate for the birth year 1992 was 45.0%.

**Table 1:** Sport-specific response rates of the quantitative study part

Kind of sport	Back	Sent out
Direct Distribution		

Archery	12	15
Athletics	134	176
Badminton	22	30
Cycling (Track cycling) Cycling (BMX cycling) Cycling (Mountain biking) Cycling (Road race)	81	141
Equitation (Dressage)	5	8
Equitation (Eventing)	6	8
Equitation (Jumping)	8	10
Fencing	22	35
Field hockey	65	101
Judo	29	42
Shooting	10	20
Swimming (Diving)	15	25
Swimming (Synchronized swimming)	8	14
Table tennis	18	24
Taekwondo	19	43
Triathlon	9	15
Volleyball (beach)	9	17
Volleyball (indoor)	46	70
Weightlifting	19	37
Alpine skiing	15	23
Biathlon	12	20
Cross-country skiing	19	24
Curling	4	4
Freestyle skiing	3	4
Ice hockey	61	109
Luge	14	24
Nordic combined	8	13
Short track speed skating	16	23
Skeleton	7	9
Ski jumping	14	17
Snowboarding	6	14
Speed-skating	44	66

Central Distribution		

Postal Reshipment		

Gymnastics (Artistic)	17	41
Gymnastics (Rythmic)	3	16
Gymnastics (Trampoline)	4	24
Soccer	42	98
Swimming	61	109

Training Courses		

Canoe/kayak (slalom)	13	14
Canoe/kayak (sprint)	19	30
Handball	61	81
Modern pentathlon	3	3
Rowing	27	27
Water polo	12	13
Figure skating	14	14

Via trainer		

Boxing	3	27
Tennis	7	26
Wrestling (Freestyle)	28	28

Mixed Distribution (Training Course AND Postal Reshipment)		

Basketball	64	111

### Central topics and data collection techniques - qualitative study

#### Qualitative study tool development

We used a mix of qualitative methods to collect data, combining participant observations with individual interviews with different actors. This methodology allowed us to account for the complexity of the phenomena, to examine different perspectives of health and nutrition within youth elite sports, and to triangulate the data. We used a range of qualitative research tools including in-depth semi-structured interviews, health-related biographical mappings, health-related network mappings, focused interviews, participant observation, and document analysis.

#### Interview guides and graphic tools

**Figure 2:**
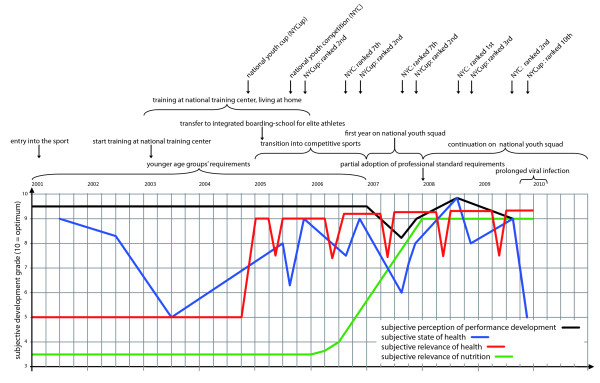
**Exemplary biographical mapping, created by an interviewed athlete**.

**Figure 3:**
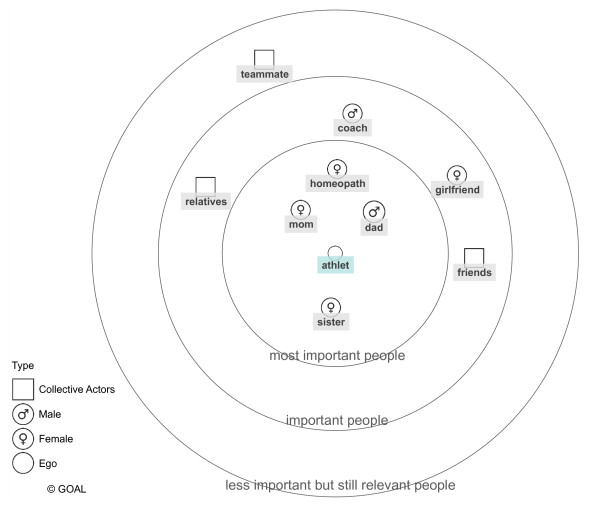
**Exemplary health-relevant network card, created by an interviewed athlete**.

We established project-specific interview guides for athletes and experts using open-ended questions allowing the interviewees to speak about their experience, knowledge and view on health and nutrition. Interview guides for the in-depth, semi-structured interviews with athletes differed only slightly, reflecting the distinctiveness of their discipline (artistic gymnastic, biathlon, handball and wrestling). For example, in the case of wrestling, we added specific questions on making weight. Furthermore, the athlete interview guides integrated two graphic tools: (1) health-related biographical mapping (Figure 2), which we developed based on earlier research projects in German elite sports [41,2,26] and (2) a health-related network mapping (Figure 3). For the biographical mapping, athletes were given a premade matrix on which they plotted points retracing their career and crucial moments for their well-being. At different moments of the interview, athletes were asked to evaluate and retrace their state of health and the relevance of health and nutrition within their biography. Drawing these personalized health-related maps, gave athletes a more playful tool to visually express their health experience. The same applied to the health-related network maps, used to ask the adolescent athletes about their health-related social networks. The ego-centered network maps [42] were generated using the software tool VennMaker 1.0 [43]. They consisted of three hierarchical circles positioned around a center point representing the athlete (ego). We asked the interviewed athletes to select people of importance to their health and to plot them within the hierarchical circles. Expert interview guides were adjusted for each sport discipline and work function within the sport (trainer, physician, physiotherapist).

We piloted the interview guides on adolescent elite athletes and experts prior to executing the study. This allowed us to adjust the question wording of and to tailor interview protocols specific to our research.

#### Participant observation and document analysis

For each discipline, we triangulated individual interviews with short-term observations during training sessions and competitions. On average, the researcher who conducted the qualitative study spent a week per discipline at German youth elite teams' national team training camps and attended one of their major national or international competitions.

Additionally, we conducted document analysis by gathering further information on key actors, our interview partners and the organizational system, both from print and online sources.

### Central topics within the qualitative interviews

#### Health and nutrition practices

Within the in-depth (semi-structured) athletes' interviews, we explored adolescent athletes' health representations and health and nutrition practices. We also investigated the athletes' ailment and injury experiences because previous research shows that the athletes' biography plays an important role in developing constructions about health. Additionally, the interviewer asked athletes about their ideas for optimal health management. At the end of the interview, we gathered demographic data and background information on the participant's current health state, using the corresponding part of the quantitative study questionnaire.

Additionally, we conducted document analysis by gathering further information on key actors, our interview partners and the organizational system, both from print and online sources.

#### Health relevant bifurcations in the biography and health-related networks

In order to trace biographic developments, to focus crises and to detect health relevant bifurcations in the interviewed athletes' biography, we integrated the aforementioned biographical mapping method (Figure 2) in athletes' interviews. The mapping process helped the interviewees to recall past events and to reconstruct biographic developments.

Additionally, we used a network map to explore and visualize the athletes' health relevant network (Figure 3).

### Data quality

#### Quantitative study

To reduce any potential desirability bias, every athlete filled in his own questionnaire alone at home or elsewhere (e.g. at a training site) and put it pseudonymized into a prepaid self-addressed envelope. We instituted this step to minimize the possibility of trainers or parents seeing the answers.

A possible limitation of our investigation was its length, with the field research lasting from February 2010 until January 2011. Yet, it is this large time span that gave us the unique possibility of obtaining a complete sample of Olympic sports athletes. Another possible problematic issue is that dependent on the sport, the respective periods of competition and training as well as the demands and stress on the individual athlete varied greatly over the year. Therefore, some athletes may have filled in the questionnaire during demanding times of competition and intense training (e.g., when the survey was conducted centrally), while others may have filled in the questionnaire during a regeneration period. It is conceivable that the time that the investigation took place within an athlete's schedule may have effected answering behavior.

When we asked for drug consumption and other behavioral aspects it is possible that respondents did not answer truthfully for reasons of social desirability. Furthermore, the question about the athletes' use of diuretics in and out of competition could prove to be problematic because the World Anti-doping Code declares diuretics as illicit drugs. We tried to compensate for any problems by including the potential answer, "I haven't done it, but I've thought about it," in order to detect the athletes' willingness to take diuretics.

#### Qualitative Study

Only one of the study's authors and a research assistant were assigned to conduct the qualitative study, which guaranteed coherence to the established procedures for interviews and participant observations. In-depth interviews varied considerably in length, lasting between 75 and 150 minutes. Focus interviews with diverse experts lasted, on average, 85 minutes. The following table (Table 2) summarizes the total collected qualitative data material, specifying sampling size and methods applied in all disciplines.

**Table 2:** Overview qualitative study data collection

	Biathlon	Gymnastics	Handball	Wrestling	Total
**In-depth interviews (total)**	**6**	**6**	**6**	**6**	**24**

*athletes (female)*	*3*	*3*	*3*	*3*	

*athletes (male)*	*3*	*3*	*3*	*3*	

**Focus interviews (total)**	**4**	**8**	**8**	**8**	**28**

*experts (female team)*		*4*	*4*	*4*	
	4				
*experts (male team)*		*4*	*4*	*4*	

**Participant observation (total in days)**	**6 + 1**	**12 + 2**	**12 + 3**	**12 +2**	**42 + 8**

*Training & competition (female team)*		*6 + 1*	*6 + 0*	*6 + 1*	
	*6 + 1*				
*Training & competition (male team)*		*6 + 1*	*6 + 3*	*6 + 1*	

* For reasons of anonymity, no further details can be given on the experts functional roles and the athletes' ages.

### Data analysis

#### Quantitative study

The data analyses of the above mentioned research questions take place in three steps. First, we carry out an univariate absolute and relative frequency count in order to obtain descriptive information on study's participants. In the second step we examine sports discipline, gender differences, and differences between athletes and athletes versus non-athletes using bivariate chi-square tests and unpaired t-tests. Besides we apply multivariate analyses in a third step using the statistical instruments of multiple linear and logistic regression analysis. Therefore, the athletes' are compared to data from the representative reference population of non-athletes (e.g., such reference data is available for KiGGS survey or the PHQ-2). In addition to these comparisons, we compare our data with other samples, for example, with a population showing eating disorders. We will carry out all tests as two-tailed tests with significance levels of p < 0.05 using the statistical analysis system, SPSS PASW Version 18.0.0 (SPSS Inc., Chicago, IL, USA).

#### Qualitative study

Interviews were recorded and transcribed verbatim using a simplified Conversation Analytic transcription system (widely used in German-speaking countries as "GAT"). This enables us to display prosodical and interactional features of the interviews within the transcripts as well as to factor this supplementary information into our analysis. Additionally we expanded the participant observation field notes into "extended accounts" [44]. When we process the interview transcripts and extended accounts, we use the text analysis software MAXQDA to process interview transcripts and extended accounts. Hermeneutic reconstructive analysis produces structured data for each athlete and discipline. We then compare the data to identify over-individual patterns and generative mechanisms.

## Discussion

The GOAL Study aims to provide new evidence about the individual health management among adolescent elite athletes. It thereby deals with a research topic, which up until now, has widely been neglected. The GOAL Study's design exhibits several strengths: First, the study consists of a quantitative survey and qualitative case studies and thus, combines multiple complementary methodological approaches. This allows for a comprehensive description and explanation of adolescent athletes' health-related problems. Second, we conducted the quantitative survey to obtain a complete sample of young German elite athletes, which ensures highly valid and representative data. Another of the study's strengths is the response rate. When compared with similar studies, GOAL Study qualified as "high rate" [45], enhancing our belief that we will indeed be able to draw valid conclusions from the large and representative data basis. Third, these conclusions will serve as a knowledge basis from which concepts can be transferred, which is also an aim of the GOAL Study. This currently developed transfer concept will provide evidence-based guidelines concerning health management for the athletes themselves and other relevant individuals (e.g. trainers). Since it will be difficult to give instructions and make recommendations for every single sport, we will at least be able to provide guidelines for sports' categories.

## Competing interests

The authors declare that they have no competing interests.

## Authors' contributions

AT, SZ and SS were the principal investigators. JM coordinated the field work of the Study. All other authors were scientific co-investigators. KD, AS and KEG conducted the quantitative part and AMS the qualitative part of the study, including field work, data management, statistical analyses and reporting. All authors read and approved the final manuscript.

## Pre-publication history

The pre-publication history for this paper can be accessed here:

http://www.biomedcentral.com/1471-2458/11/410/prepub
